# Toward a comprehensive view of cancer immune responsiveness: a synopsis from the SITC workshop

**DOI:** 10.1186/s40425-019-0602-4

**Published:** 2019-05-22

**Authors:** Davide Bedognetti, Michele Ceccarelli, Lorenzo Galluzzi, Rongze Lu, Karolina Palucka, Josue Samayoa, Stefani Spranger, Sarah Warren, Kwok-Kin Wong, Elad Ziv, Diego Chowell, Lisa M. Coussens, Daniel D. De Carvalho, David G. DeNardo, Jérôme Galon, Howard L. Kaufman, Tomas Kirchhoff, Michael T. Lotze, Jason J. Luke, Andy J. Minn, Katerina Politi, Leonard D. Shultz, Richard Simon, Vésteinn Thórsson, Joanne B. Weidhaas, Maria Libera Ascierto, Paolo Antonio Ascierto, James M. Barnes, Valentin Barsan, Praveen K. Bommareddy, Adrian Bot, Sarah E. Church, Gennaro Ciliberto, Andrea De Maria, Dobrin Draganov, Winson S. Ho, Heather M. McGee, Anne Monette, Joseph F. Murphy, Paola Nisticò, Wungki Park, Maulik Patel, Michael Quigley, Laszlo Radvanyi, Harry Raftopoulos, Nils-Petter Rudqvist, Alexandra Snyder, Randy F. Sweis, Sara Valpione, Lisa H. Butterfield, Mary L. Disis, Bernard A. Fox, Alessandra Cesano, Francesco M. Marincola, Society for Immunotherapy of Cancer (SITC) Cancer Immune Responsiveness Task Force and Working Groups

**Affiliations:** 1 Sidra Medicine, Doha, Qatar; 2 AbbVie, Redwood City, CA USA; 3 Department of Radiation Oncology, Weill Cornell Medical College, New York, NY USA; 4 Sandra and Edward Meyer Cancer Center, New York, NY USA; 5 Université Paris Descartes/Paris V, Paris, France; 6 The Jackson Laboratory for Genomic Medicine, Farmington, CT USA; 7 Koch Institute for Integrative Cancer Research at MIT, Cambridge, MT USA; 8 NanoString Technologies, Inc., Seattle, WA USA; 9 Perlmutter Cancer Center, New York Langone Health, New York, NY USA; 10 University of California, San Francisco, San Francisco, CA USA; 11 Memorial Sloan Kettering Cancer Center, New York, NY USA; 12 Oregon Health & Science University, Portland, OR USA; 13 Department of Medical Biophysics, Princess Margaret Cancer Centre University Health Network, University of Toronto, Toronto, Canada; 14 Washington University School of Medicine in St. Louis, St. Louis, MO USA; 15 INSERM, Laboratory of Integrative Cancer Immunology, Equipe Labellisée Ligue Contre le Cancer, Sorbonne Université, Sorbonne Paris Cité, Université Paris Descartes, Université Paris Diderot; Centre de Recherche des Cordeliers, F-75006 Paris, France; 16 Massachusetts General Hospital, Boston, MA, USA and Replimune, Inc., Woburn, MA USA; 17 Perlmutter Comprehensive Cancer Center, New York University School of Medicine, New York University Langone Health New York, New York, NY USA; 18 UPMC Hillman Cancer Center, University of Pittsburgh, Pittsburgh, PA USA; 19 University of Chicago, Chicago, IL USA; 20 Abramson Family Cancer Research Institute, University of Pennsylvania, Philadelphia, PA USA; 21 Yale School of Medicine, New Haven, CT USA; 22 The Jackson Laboratory Cancer Center, Bar Harbor, ME USA; 23 R. Simon Consulting, Potomac, MD USA; 24 Institute for Systems Biology, Seattle, WA USA; 25 University of California, Los Angeles, Los Angeles, CA USA; 26 MedImmune, Gaithersberg, MD USA; 27 Istituto Nazionale Tumori-IRCCS Fondazione ‘G. Pascale’, Naples, Italy; 28 Stanford University, Stanford, CA USA; 29 Rutgers University, New Brunswick, NJ USA; 30 Kite, a Gilead Company, Santa Monica, CA USA; 31 IRCCS Istituto Nazionale Tumori Regina Elena, Rome, Italy; 32 Università degli Studi di Genova and Ospedale Policlinico San Martino IRCCS, Genoa, Italy; 33 Calidi Biotherapeutics, San Diego, CA USA; 34 Department of Neurosurgery, Division of Pediatric Neurosurgery, Primary Children’s Hospital, University of Utah, Salt Lake City, UT USA; 35 Department of Radiation Oncology, Icahn School of Medicine at Mount Sinai, New York, NY USA; 36 Lady Davis Institute for Medical Research, Jewish General Hospital, Montreal, QC Canada; 37 Caprion Biosciences Inc., Montreal, QC Canada; 38 Bristol-Myers Squibb Company, Redwood City, CA USA; 39 Ontario Institute for Cancer Research, Toronto, Ontario Canada; 40 Bayer HealthCare Pharmaceuticals Inc., Whippany, NJ USA; 41 Merck & Co., Kenilworth, NJ USA; 42 CRUK Manchester Institute and The Christie NHS Foundation Trust, The University of Manchester, Manchester, UK; 43 Parker Institute for Cancer Immunotherapy, San Francisco, CA USA; 44 University of Washington, Seattle, WA USA; 45 Earle A. Chiles Research Institute, Robert W. Franz Cancer Center, Providence Cancer Institute, Portland, OR USA; 46 Refuge Biotechnologies Inc., 1505 Adams Drive, Suite D, Menlo Park, CA 94025 USA

**Keywords:** Cancer immune responsiveness (CIR), Immune checkpoint inhibitor (ICI), Immune oncology (IO), Immunotherapy, Tumor microenvironment (TME), Tumor mutational burden (TMB), Immunogenic cell death (ICD), Biomarker, Germline molecular alterations, Somatic molecular alterations, Cancer immune phenotype

## Abstract

Tumor immunology has changed the landscape of cancer treatment. Yet, not all patients benefit as *cancer immune responsiveness (CIR)* remains a limitation in a considerable proportion of cases. The multifactorial determinants of CIR include the genetic makeup of the patient, the genomic instability central to cancer development, the evolutionary emergence of cancer phenotypes under the influence of immune editing, and external modifiers such as demographics, environment, treatment potency, co-morbidities and cancer-independent alterations including immune homeostasis and polymorphisms in the major and minor histocompatibility molecules, cytokines, and chemokines. Based on the premise that cancer is fundamentally a disorder of the genes arising within a cell biologic process, whose deviations from normality determine the rules of engagement with the host’s response, the *Society for Immunotherapy of Cancer (SITC)* convened a task force of experts from various disciplines including, immunology, oncology, biophysics, structural biology, molecular and cellular biology, genetics, and bioinformatics to address the complexity of CIR from a holistic view. The task force was launched by a workshop held in San Francisco on May 14–15, 2018 aimed at two preeminent goals: 1) to identify the fundamental questions related to CIR and 2) to create an interactive community of experts that could guide scientific and research priorities by forming a logical progression supported by multiple perspectives to uncover mechanisms of CIR. This workshop was a first step toward a second meeting where the focus would be to address the actionability of some of the questions identified by working groups. In this event, five working groups aimed at defining a path to test hypotheses according to their relevance to human cancer and identifying experimental models closest to human biology, which include: 1) Germline-Genetic, 2) Somatic-Genetic and 3) Genomic-Transcriptional contributions to CIR, 4) Determinant(s) of Immunogenic Cell Death that modulate CIR, and 5) Experimental Models that best represent CIR and its conversion to an immune responsive state. This manuscript summarizes the contributions from each group and should be considered as a first milestone in the path toward a more contemporary understanding of CIR. We appreciate that this effort is far from comprehensive and that other relevant aspects related to CIR such as the microbiome, the individual’s recombined T cell and B cell receptors, and the metabolic status of cancer and immune cells were not fully included. These and other important factors will be included in future activities of the taskforce. The taskforce will focus on prioritization and specific actionable approach to answer the identified questions and implementing the collaborations in the follow-up workshop, which will be held in Houston on September 4–5, 2019.

## Background

Tumor immunotherapy has changed the therapeutic landscape for patients with cancer. While several classes of drugs are demonstrating clinical benefit, *immune checkpoint inhibitor (ICI) therapy* in particular has received considerable attention because these agents improve overall survival and are effective in a wide range of tumor types [1, 2]. Why some patients respond initially to ICI therapy and not other *immuno-oncology (IO)* regimens is not clearly understood. Indeed, many cancer patients do not benefit from IO treatments even when the tumors display favorable immune characteristics [3] and the reason(s) for their resistance to these approaches remain(s) uncertain. To date, established experimental systems have been flawed in answering this critical question because they cannot adequately replicate the complicated evolutionary processes inherently impacting human cancers in immune competent hosts. While current models are useful for hypothesis generation, they need to be realigned and reinterpreted within the framework of human biology. Thus, a cohesive blueprint is needed to generate definitive information relevant to human cancer. This is why the *Society for Immunotherapy of Cancer (SITC)* organized a Task Force on *Cancer Immune Responsiveness* (*CIR*) to stimulate interactions among multiple disciplines and outline salient open questions and define new priorities for research in tumor immunology and immunotherapy [4].

The taskforce was launched by a workshop held in San Francisco on May 14–15, 2018 that convened immunologists, geneticists, cell biologists, molecular biologists, biophysicists, computational analysts and oncologists, and aimed at two preeminent goals: 1) to identify the fundamental questions related to CIR and 2) to create an interactive community of experts that could guide scientific and research priorities by forming a logical progression supported by multiple perspectives to answer the fundamental questions and uncover mechanisms of CIR.

Diverse and often divergent observational or experimental justifications for immune resistance have been described [5, 6]. Indeed cancers can be conceptually distinguished into immune “active” versus immune “silent” tumors according to the transcriptional expression of a set of genes termed the *immunologic constant of rejection* (*ICR*) [7, 8] that defines the continuum of cancer immune surveillance within the *tumor microenvironment* (*TME*) [9]. Galon et al. have shown that the immune active or immune silent tumors associated with cytotoxic and memory T-cells, Th1 cells, and interferon-gamma (IFN-γ) signature are correlated with long-term survival or rapid recurrence respectively [10, 11]. The consensus Immunoscore categorizing inflamed and non-inflamed tumors was recently validated globally with profound clinical implications [12]. For instance, the characterization of primary colon cancer immune infiltrates by the Immunoscore could refine and extend the proportion of Stage IV patients eligible for immune checkpoint inhibitors treatment, as suggested by Le et al. [13, 14]. Furthermore, the Immunoscore was able to identify good prognostic colon cancer Stage II patients with high-risk clinico-pathological features for whom adjuvant treatment may be avoided, underlying once more its clinical utility [15]. In locally advanced Stage III colon cancer, risk assessment and more precise risk categories should be used to inform the duration of adjuvant chemotherapy. The consensus Immunoscore stratifying patients with stage III colon cancer could help aid in clinical decision-making, in particular the possibility to decrease un-necessary chemotherapy regimen within high Immunoscore patients [12, 16–18].

The 4-category classification of tumors (immune hot, altered-immunosuppressed, altered-excluded, and cold) based on their immune infiltrates and Immunoscore proposed in 2009 [11] could prove instrumental in guiding the most appropriate therapeutic approach [19]. Ayers et al. [3] have categorized tumor types according to an analytically and clinically validated IFN-γ-related gene signature termed the *tumor inflammation signature* (*TIS*) that largely overlaps with the ICR and importantly is predictive of clinical benefit of ICI therapy. Other signatures outlining the central role that IFN-γ signaling plays in determining the immune landscape of cancer and its responsiveness to immunotherapy agents have been described [6, 9, 20–22]. Of note, as recently reported by Cristescu et al. shown in *The Cancer Genome Atlas* (TCGA) dataset, a strong correlation (*r* > 0.9) between several other previously published transcriptional signatures reflective of the T cell-inflamed TME associated with cytolytic processes was demonstrated [23].

A set of about one thousand breast cancers from TCGA is subdivided into separate immune phenotypes called ICR-1 to ICR-4 according to the incremental level of expression of ICR genes. The expression of transcriptional signatures associated with immune regulatory properties is then considered for their presence in each immune phenotype [6]. The transcriptional signatures are representative of distinct immune regulatory mechanisms including the immune checkpoint cluster [24], regulatory T cells [25], IL-23/IL-17 axis [26], myeloid suppressor cells [27], IDO [28], *immunogenic cell death* (*ICD*) [29], TAM tyrosine kinase receptors [30], hypoxia [31], cancer-associated fibroblasts [32] and barrier molecules [33]. Self-organizing clustering distributes signatures according to the immune landscapes pre-defined by the ICR signature and demonstrates that most immune regulatory functions pertain to immune active cancers (ICR-4) [6]. Most recently, it has been reported that tumors with a high glycolytic rate are resistant to *adoptive cellular therapy* (*ACT*) suggesting that modulation of metabolic pathways may also affect immune cell function [34]. It has also been shown in humans that TME with increased metabolism (not necessarily and only associated with increased glycolysis) are resistant to immune checkpoint blockade including *programmed cell death protein 1* (*PD-1*) blockade [35, 36]. However, the actual rate and frequency of metabolic alterations either related to intrinsic immune cell function or reactive to hypoxic conditions in different cancer types are currently unknown.

Enrichment of immune regulatory functions within the active immune landscapes [6] suggests that resistance to ICI therapy is due to the co-existence of alternate regulatory mechanisms that overrule a single blockade. We refer to this mechanism as *adaptive immune resistance* and propose that the cancer immunity cycle described by Chen and Mellman [37] pertains particularly, and perhaps exclusively, to this context. Conversely, immune silent cancers are unlikely to respond to ICI therapy because checkpoints are irrelevant to their evolution (*primary immune resistance*). In addition, immune responsive tumors may become resistant under the selective pressure induced by therapy by developing escape mechanisms (*acquired immune resistance*). Finally, a non-biological type of resistance to therapy (*pseudo immune resistance*) should be specified that it occurs when a given treatment is terminated prematurely in order to limit toxicity. Similarly, other external factors may be at the basis of lack of response that are beyond the biology of individual tumors such as variations in product quality in the case of ACT [38] or in cases of immunodeficiency in which patients seem unable to mount an effective immune response, despite having favorable predictors at the tumor site [39].

Immune active cancers display a distinct genetic profile characterized by a high mutational burden [40, 41]. This association is commonly attributed to increased stochastic chances of expressing neo-epitopes that stimulate adaptive nonself-recognition [42, 43]. In immune active cancers, enriched mutations affect the function of cancer driver genes, leading to the hypothesis that cancer evolution in the immune-competent host faces a stochastic binary choice: some cancers accrue an orderly succession of genetic alterations that engender essential growth advantages in strict avoidance of additional unnecessary functions; this process may be compared to the assembly of normal tissues orchestrated by differentiating stem cells during development [44]. The mutational profile characteristic of immunogenic tumors is in contrast with the higher frequency of copy number alterations observed in immune silent tumors. In this case, an inverse correlation with immune signatures or immune infiltration has been observed [45, 46]. As soon as deviations occur from this orderly process and cancer growth becomes dependent predominantly on genetic instability, a “*trial-and-error*” reshuffling of genetic traits selects for a proliferative advantage over normal cell growth. Pediatric tumors arise with primary genetic instability and most adult tumors with secondary genetic instability associated with prolonged stress and inflammation. The intrinsic biology of the cancer cell primarily orchestrates its surroundings [47] by releasing factors that stimulate stromal and vascular architecture in the developing new tissue as per Virchow’s “*healing wound*” model [7, 48]. The cross-talk with cells may result in chemo-attraction of innate and adaptive immune cells turning cancer into a chronically inflamed tissue [48]. This disorderly process, however, appends the stochastic risk of gradually accumulating unnecessary functions such as chemo-attraction that may trigger immune recognition [48]. In addition, it is possible that genetic instability may result in a disorderly cell cycle prone to ICD [49]. Indeed, the expression of the ICD signature is tightly associated with the immune active landscape [6, 50] characterized in turn by genetic instability [51]. Thus, the destabilization of the cellular life cycle resulting in ICD may represent the primary trigger of immunogenic reactions in line with Polly Matzinger’s danger model [52] associated with release of *damage associated molecular pattern* (DAMP) [53, 54].

CIR is determined by the summative effects of the genetic background of the host, somatic alterations related to the oncogenic process, and environmental modifiers [55]. This synopsis will present various views on how these determinants may affect immune responsiveness and offer an integrated and sequential view. We appreciate that this first effort is far from comprehensive and that other relevant aspects related to immune responsiveness such as the microbiome and the metabolic status of cancer and immune cells are not included in our initial assessment. These and other important factors as well as addressing the relevant questions from each working group will be included in future activities and meetings of the taskforce.

## Germline genetic contributions to CIR

Growing evidence suggests that the host immunity is influenced by inherited factors. However, the germline genetic contribution to CIR has not been systematically explored due to several hurdles. First, since highly effective immunotherapeutic approaches such as the ICIs have been only recently implemented, limited datasets are preventing conclusive association studies. Second, large datasets such as TCGA have scant information on clinical outcome, particularly on response to IO agents. In addition, TCGA collected samples from primary tumors (with the exception of melanoma) while IO is applied mostly in metastatic settings. Yet, these datasets are still useful for assessing the contribution of the genetic background to the development of anti-tumoral immunity by matching germline data to the functional orientation of TME derived from transcriptional data.

### Rationale supporting the relevance of germline studies to CIR

Twin have shown that both heritable and non-heritable factors significantly contribute to an observed phenotype [56–58]. These findings might have important implications for CIR as divergent baseline conditions might reflect a different predisposition to mount an adequate immune response after a certain stimulus.

*Genome-wide association studies* (*GWAS*) have identified more than three hundred susceptibility loci predisposing to the development of autoimmune diseases. Moreover, studies of severe autoimmune or immunodeficiency syndromes identified several causative variants [59]. Polymorphisms of *Human Leukocyte Antigen* (*HLA*) molecules have been associated with variable responses to infection, inflammatory conditions, autoimmune diseases, and development of virus-induced tumors and some not-known to be virus-induced such as non-small cell lung cancer [60] and hematological malignancies [61]. However, GWAS are limited to finding common variants. Whole exome sequencing (WES) offers complementary information to GWAS on rare variants in coding regions, and ultimately, whole genome sequencing (WGS) can systematically assess common and rare variants, as well as structural variation.

As compared to common risk polymorphisms, which are mostly localized in non-coding regions, rare protein-coding variants are more likely to lead to a loss or altered function of the protein [59] that may influence treatment outcome in patients who do not demonstrate otherwise signs of immune dysfunction(s). This strategy also might be explored to gain mechanistic insights about severe immune-related adverse events. It is possible that the germline control of host immunity is not subjected to the selection pressure relevant to the general population in relation to infectious challenges. In contrast to GWAS designs of disease risk, which yielded only the low-penetrant risk effect associated with tested common genetic variants, these effects may be substantially enhanced in the context of immunotherapy treatments. Thus, minor alleles of common variants may be comparably distributed in the population but under iatrogenic immune stimulation their effect on cancer responsiveness can become noticeable. Therefore, empirical validation of the contribution of common variants to CIR will be needed as part of larger genome-wide scans, including IO-based GWAS. For example, a recent study of melanoma showed that the functionally relevant common single nucleotide polymorphisms in interleukin pathways may associate with improved melanoma survival independent of the other prognostic predictors [62].

### Potential mechanisms implicated in germline immune modulation

**Fig. 1:**
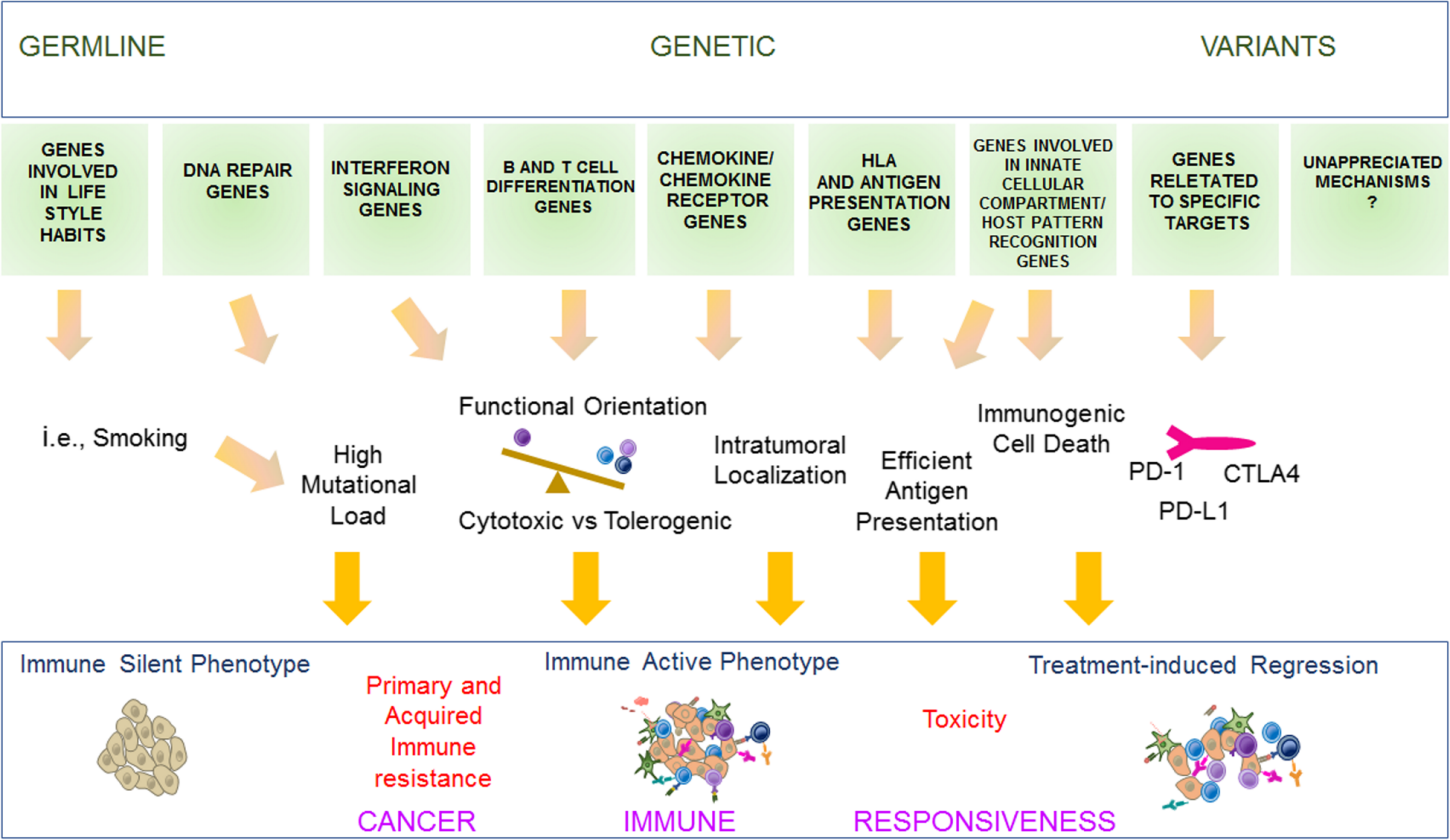
Germline contributions to CIR. Germline genetic contributions to CIR. Genetic germline variants can influence CIR in different ways, which are tightly interconnected. Variants associated with attitude to smoke or mutation in DNA-repair genes (e.g., DNA mismatch repair genes) can cause the accumulation of somatic alterations which in turn might facilitate the parallel development of neoepitope-mediated immune recognition.. Polymorphisms of genes that modulate critical immunologic pathways such as IFN signaling and differentiation and function of T cells and B cells might influence the development of tolerant vs cytotoxic TME. The same could be said of variants in genes governing antigen presentation such as HLA class I and II, ICD, innate-immunity function in macrophages, *natural killer* (*NK*) cells, and granulocytes. Polymorphisms of TLR4, P2RX7, and FPR1 have been associated with differential outcome in breast and colon cancer patients treated with adjuvant chemotherapy, likely through the modulation of ICD-mediated anti-tumor immune response [63, 64]. HLA-E, a non-classical HLA molecule, is recognized by specific NK cell lectin-type receptors with either activating or inhibiting activity in the context of specific and redundant antigenic presentation. HLA-E polymorphisms might have an impact on anti-tumor response independently from the CIR mechanisms recognized so far [65]. Variants in genes encoding for chemokines or chemokine receptors might also differentially modulate intra-tumoral recruitment of immune cells. Variations in protein-coding regions of genes affecting structure or expression of molecules targeted by IO agents might influence their efficacy. Polymorphisms of *crystallizable fragment* (*Fc*)-γ receptor genes have been associated, although inconsistently, with distinct outcomes in patients treated with Rituximab and Trastuzumab [66]. Such variations might potentially influence the efficacy ICIs via *antibody-dependent cytotoxicity* (*ADCC*) lysis of target or tumor cells [67]

Germline genetic factors might influence CIR in myriad ways [63–67]. Some examples are shown in Fig. 1. In addition to variants of immune-related genes, mutations of DNA-repair genes can cause accumulation of somatic alterations by increasing genomic instability, which in turn might facilitate the development of neoepitope-mediated tumor rejection. It is likely that high *tumor mutational burden* (*TMB)* contributes to responsiveness to ICIs in patients with germline mutations of mismatch-repair. Would patients carrying such mutations be also more likely to develop acquired, immune resistance? The influence of germline variants on cancer-cell’s intrinsic features to modulate anti-tumor immune response needs to be addressed by germline-somatic integrative analyses through whole-exome/whole-genome sequencing [68] paired with clinical outcome information. Mixed responses are common in ICI-treated patients and are largely attributed to somatic tumor genomic heterogeneity [69]. Can the germline genetic background modify the degree of tumor immune heterogeneity and therefore the likelihood to develop mixed responses?

### Evidence supporting the existence of a link between germline variants and CIR and clinical implications

Few studies have investigated the germline contributions to CIR. In metastatic melanoma, the link between polymorphisms of genes implicated in the pathogenesis of autoimmune diseases, such as *C-C motif chemokine receptor* (*CCR*)-5 and *IFN regulatory factor*-5 and responsiveness to chemo-immunotherapy [70] or adoptive therapies [71, 72], was reported. Several studies indicated that CTLA-4 polymorphisms affect response to CTLA-4 blockade [73–75]. In all cases, the reports are inconclusive since these studies lacked adequate validation. A tenuous or lack of associations between HLA polymorphisms and responsiveness to the systemic administration of *interleukin* (*IL*)-2 in metastatic melanoma was reported by early studies [76], while a modest, yet significant, association between HLA variants and survival was detected in melanoma patients treated in adjuvant settings with IFN-α [77]. A recent study testing the effect of immunomodulatory expression quantitative trait loci (eQTLs) identified an association between eQTL in IL-10/BATF3 locus on 1q32 and survival in melanoma, complementing other established clinico-pathological prognostic markers [62]. Interestingly, the associated eQTL is a proxy of variants associated with multiple autoimmune conditions [78], suggesting that propensity to autoimmunity provides survival advantage in immunogenic tumors.

Recently, large genetic study on melanoma and lung cancer patients treated with ICIs has shown that a low level of germline HLA-I heterozygosity is strongly associated with poor outcomes [79]. This effect is enhanced by, but is not dependent on TMB. Furthermore, efficacy of ICI was diminished by somatic loss of HLA-I heterozygosity by the tumor cells. Molecular dynamics simulations of HLA super-types associated with poor prognosis revealed distinctive elements that might affect neoantigen recognition by cytotoxic T cells [79]. Preliminary studies in melanoma patients employing WES and genotyping suggest that genetic variants in interleukin- and chemokine-related pathways are associated with differential responsiveness and toxicity across anti-PD-1 and anti-CTLA-4 treatments [80]. Also, germline mutations disrupting miRNA regulatory pathways have been linked to toxicity and responsiveness to PD-1 blockade [81]. Interestingly, recent data suggests that polymorphisms of Fc-γ receptor influence the efficacy of anti-CTLA-4 treatment, and the modulation seems to be relevant only in the context immunologically active tumors [67]. Preliminary data generated by the analysis of TCGA samples suggests that a proportion of transcriptional signatures related to leukocyte abundance and functional orientation in the TME is partially heritable (E. Ziv, this workshop). In addition, the expression of several immune-related genes and immune-related signatures have been recently associated with different germline variants [82].

The identification of common genetic variants associated with treatment outcome might lead to the development of better patient stratification. If such variants are identified, polygenic scores might be used to define predictive classifiers. While it is unlikely that germline variants can be used as a single marker for stratification purposes in metastatic patients, they can be integrated with other biomarkers (such as mutational load, presence of somatic mutations, or transcriptional and morphologic features) to develop multi-factorial predictors. In the adjuvant setting, germline variants associated with toxicity can guide the selection of patients for the modest survival benefit. Additionally, germline testing may inform the assessment of therapeutic index for administering immunotherapies in patients with pre-existing autoimmune diseases.

Germline investigations might also have profound implications for therapeutic interventions. For example, the observation that specific HLA-I super types are associated with increased immune responsiveness [79] offers the opportunity to develop specific vaccination strategies targeting immune dominant, super type-restricted neoantigens. Similarly, the associations of eQTLs in IL-10 locus with melanoma progression [62] may expand the applicability to immunotherapy with the concurrent targeting of IL-10 receptor during the treatment with ICI [83, 84]. It will be critical to understand how mutational signatures across cancer types and the HLA-I genotype of patients interact to impact the repertoire of neoepitopes presented by tumor cells, and affect lymphocyte density, immune contexture, CIR and ultimately clinical outcome.

### Controversies

While genetic studies may be helpful, each approach has significant limitations. GWAS studies are limited to identifying common variants which are either directly genotyped on an array or, more commonly, imputed. Although GWAS have identified a large number of loci associated with autoimmune disorders and other complex traits, for most complex phenotypes, discovered loci accounted for a relatively small fraction of the heritability of the phenotype. The effect sizes of the vast majority of these variants are small, with the vast majority of these odds ratios being 1.2 or less [85], with few notable exceptions showing the stronger effect size with meaningful utility, such as HLA effects on autoimmunity [86]. As individual markers, these variants provide limited clinical utility. However, if enough can be combined (e.g. by computing polygenic scores), they may become useful [87].

GWAS in the context of immunotherapy, as mentioned earlier, may not follow the pattern of expected low-penetrant risk effects in disease risk. Thus, it is possible that IO GWAS will identify genetic loci with stronger risk effects and clinical applicability, as suggested by recent pharmacogenomics associations [62, 88]. Importantly, the clinical risk effects of germline variations by GWAS can be enhanced by the combined testing of common and rare variation, further improving their predictive capacity [87]. While *next-generation sequencing* (*NGS*) offers notable advantages, there are also limitations: WES, as cost-efficient alternative mapping germline variations in coding regions, targets only ~ 1–2% of the genome. Nevertheless, a focused application of WES and targeted panels is widely used in screening of germline mismatch repair mutations as predictive surrogates of outcomes to PD-1 inhibitors in colon cancer [89] and other tumor types [90]. WGS provides the most comprehensive platform for germline screening in IO and CIR. However, due to the complexity of the data analysis, complementing approaches are needed, including the combined analysis of common and rare variation in gene-burden tests integrated with other layers of biological information, to aid in prioritization of non-coding but functionally-relevant germline markers.

### Take-home messages and challenges for germline genetic contributions to CIR

- Recent findings suggest that germline variants might shape intra-tumoral immune response, and influence responsiveness and toxicity to immunotherapy.
- Current large cancer databases are useful resources to explore the relationship between individuals’ genetic background and intra-tumoral immune response but lack information on treatment outcome, especially on immunotherapeutic agents.
- The constitution of appropriate databases paired with high-powered studies are needed to define the magnitude of genetic germline contributions to CIR and to identify putative germline genetic immune biomarkers.
- It is critical to establish dedicated large collaborative consortia or networks collecting harmonized clinico-pathological information, which represents a major roadblock in the systematic exploration of the germline component in IO.
- Germline information should be integrated with phenotypic information such as somatic alterations, epigenetic and transcriptional features to increase prediction accuracy.
- Analytic integrative pipelines need to be implemented for deciphering causal associations and for prioritizing putative functional variants and pathways.
- Once identified, genetic germline biomarkers might be used to increase treatment outcome, adverse event prediction and to define novel therapeutic strategies.

### Unanswered questions for germline genetic contributions to CIR and strategies to meet the challenges

- Which are the key molecular mechanisms involved in anti-tumor immunity that might be modulated by germline genetic variants?
- Are common genetic polymorphisms associated with a differential spontaneous or treatment-induced anti-tumor immune response?
- How can we implement the study of host genetic diversity to identify novel biomarkers of responsiveness or toxicity to cancer immunotherapy?

Large cooperative clinical trial groups might be best poised to accrue the necessary extremely large sample size. Thus, it is difficult to implement this approach in the therapeutic setting. As large sample sizes are needed for germline studies, *the taskforce members agree that it is critical to establish collaborative networks dedicated to these investigations*, which will allow harmonized collection of clinic-pathological information. Collaborations might occur by sharing patient samples and/or data. The cohorts could be enriched for exceptional responders, rapid “progressors”, or for patients experiencing severe adverse events. Collection of germline DNA should be included in clinical trials. Ethical and regulatory issues might represent an obstacle for sharing germline data and need to be prospectively taken into account at the time of study design. Funding agencies are often requiring to deposit in publicly accessible repositories germline and phenotypic information generated by the awarded researches, and therefore it is expected that amount of germline data for CIR exploration will increase in the next few years. National large-scale initiatives, e.g. the UK Biobank, might represent additional resources for this kind of exploration. Going forward, it would be critical to integrate germline data with phenotypic attributes, such as transcriptomic signatures, epigenetic, and somatic alterations to increase CIR prediction accuracy [91]. It is possible that the effect of some germline polymorphisms is restricted to certain cancer immune phenotypes, therefore increasing the complexity of the analytic approach [67].

To properly address the germline questions, it will be important to standardize platforms and methodological approaches. The implementation of bioinformatics pipelines, such as fine mapping strategies to prioritize putative functional variants and to identify true causal associations, will be critical [92, 93]. Direct genome-editing techniques, e.g. CRISPR/Cas9 and in vivo model for studying CIR, will offer the opportunity to translate association-study based information into biological relevant knowledge [93].

## Somatic genetic contributions to CIR

The accumulation of different genetic and epigenetic alterations are the origin of inter- and intra-tumor heterogeneity impacting cancer pathways, driving phenotypic variation, and posing significant challenges to personalized cancer medicine [47, 94, 95]. Beyond these effects, an open question in IO is whether and how tumor intrinsic features affect the characteristic of the TME. The need to address this question arises from improvements, in terms of clinical outcomes, to therapeutic approaches targeting immune cells especially in melanoma and lung cancer but also other cancers [90, 96]. Our poor understanding of the genetic mechanism contributing to the host-tumor interaction limits further development of more effective treatments. Many sources of evidence have recently shown that tumor cell-intrinsic signaling pathways and gene regulatory networks play a fundamental role in the degree of T cell infiltration [8, 97, 98]. However, the understanding of the complexity of the tumor-host interaction also requires taking into account the specific genetic makeup of the host (patient genotypes) [99] and interaction with the environment [99, 100]. In addition, mutations driven by immunologic selection have been described such as alterations in JAK [101] and IFNGR [102] genes. While knowledge about the somatic genetic contributions to the determination of immune responsiveness remains limited, future attempts aimed at addressing the above questions should utilize advanced system genetic approaches leveraging the availability of multi-omics, large-scale datasets [103].

### Predictive biomarkers of ICI therapy

The most pressing clinical question for ICI therapy is what are the predictive biomarker(s) of response and resistance to ICI therapy? Identification of such predictive biomarker(s) would improve patient selection, limit toxicity (including financial toxicity), and guide biology-based combinations thus moving the immunotherapy field towards personalized medicine [104]. In order to identify these predictive biomarkers, understanding genomic characteristics underpinning tumor immunogenicity is essential as it would enable deeper comprehension of tumor intrinsic mechanisms of primary resistance to ICI therapy (which is applicable to majority of patients treated with ICI therapy) and those mechanisms governing acquired resistance.

This critical question is currently under intense investigation. Accumulating evidence supports the existence of tumor intrinsic features such as TMB, correlated to an immune active TME and predictive of response to PD-1/PD-L1 blockade, independent of PD-L1 expression [40, 90, 105]. The use of TMB as a clinical predictive biomarker poses, however, some challenges in terms of harmonization and standardization. For example, the use of NGS panels needs the selection of suitable TMB cutoffs. Moreover, the selection of such cutoffs should take into account predictive power and specificity within different tumor indications, sequencing methodologies and depth of coverage. The assessment of biomarkers correlated to TMB such as genomic alterations in *DNA damage response* (*DDR*) genes has also been recently suggested [106]. Indeed, alterations in DDR pathways may result in higher TMB and neoantigen load, and could potentially be a more accurate predictive marker to identify ICI therapy responders. Yet, it is not clear if all DDR pathway alterations will impart increased tumor immunogenicity, and additional work is required to understand what is the impact of co-DDR pathway alterations on clinical outcomes to ICI therapy [107]. This understanding would improve patient selection strategy as well as identify ICI therapy combination therapies that may exploit these tumor-intrinsic characteristics. In addition, blood-based genomic assays with *cell free DNA* (*cfDNA*) or *circulating tumor cells* (*CTC*) assessing DDR genes as a surrogate to estimating TMB could result in a potential solution to limitations of tissue availability.

### Associations between somatic alteration and features of TME

Recent observations have demonstrated that specific somatic alterations in tumor cells correlate with changes in the TME, including overall lymphocytic content, cellular composition, and intracellular signaling [47, 108]. In some cases, functional interactions mediating these associations have been established. As highlighted previously, correlations are seen with the overall extent of DNA alterations, both mutation and copy-number alteration burden, but somatic alteration in individual proteins or pathways also impacts the TME. For example, somatic mutations that lead to tumor-intrinsic β-catenin activation have been identified as mediating exclusion of T cells from the TME [108]. In prostate cancer, KRAS^G12D^ mutations lead to increased production and secretion of *granulocyte-macrophage colony-stimulating factor* (*GM-CSF*) and accumulation of immunosuppressive myeloid cells, suppressing cytotoxic lymphocytes [109]. In glioma, *isocitrate dehydrogenase 1* (*IDH1*) mutations are associated with variations in the degree of leukocyte infiltrate, macrophage content, and repression of tumor-associated immune responses [110]. Analysis of cancer genomics has yielded a wealth of associations between somatic alterations and immune response, including with TME composition and response to ICI therapy [103, 111]. In addition, several computational studies have demonstrated strong associations between the genetic makeup of tumors and their immune contexture. By mining large scale datasets, they revealed that expression of genes associated with cytotoxic immune activation was correlated with specific mutations such as in PIK3CA or MET. However, many open questions exist on the mechanisms by which cancer-intrinsic properties affect the heterogeneity of their immune landscape, and the interrogation of the spatiotemporal regulation of the immune microenvironment requires novel in vivo genetic experimental platforms able to model concurrently the heterogeneity of the cancer cell and their crosstalk with the immune microenvironment [98].

### Associations of epigenomic alterations to the features of TME

The chromatin provides the physical substrate where epigenetic mechanisms and signaling pathways converge to coordinate transcriptional programs, playing a critical role in cellular phenotype and cellular memory. The chromatin also plays an essential role to repress transposable elements. During cancer development, the global chromatin landscape in cancer cells becomes deregulated, as a consequence of altered transcriptional profiles or mutation in genes encoding chromatin-remodeling factors, histone modifying enzymes, and DNA methylation enzymes.

This deregulated chromatin landscape of cancer cells can play a critical role in the immune landscape and immune responsiveness. As one example, SWI/SNF chromatin remodeling complex genes are inactivated through mutation in ~ 20% of cancers. One subunit of this complex, PBRM1 is mutated in ~ 40% of patients with clear cell renal cell cancer (ccRCC). PBRM1 was recently shown to inhibit activation of IFN-stimulated genes in cancer cells in response to IFN-γ produced by T cells. Indeed, PBRM1 inactivation increases sensitivity of cancer cells to T cell-mediated killing and truncating loss-of-function mutations in PBRM1 is associated with increased response rates to anti-PD-1 or anti-PD-L1 in ccRCC patients [112, 113].

Moreover, deregulated chromatin landscape in cancer cells can be targeted therapeutically to increase immune responsiveness. For example, DNA demethylating drugs were previously shown to re-activate human endogenous retrovirus (HERVs), leading to formation of dsRNA and activation of pattern recognition receptors, such as the MDA5/MAVS anti-viral pathway. This leads to a ‘viral mimicry’ state, where cancer cells activate antiviral responses, leading to immunogenic cell death, Type I and III IFN production, and increased antigen processing and presentation [114, 115]. Recently, reactivation of retroviruses have been associated with heightened response to checkpoint blockade in patients with renal cancer [116, 117].

Finally, besides cancer cell intrinsic chromatin deregulation, immune cells in the TME can also show epigenetic alterations. A recent study showed that chronically stimulated T cells acquire de novo DNA methylation programs that lock them into an exhausted phenotype. Moreover, inhibition of DNA methyltransferases can avoid the onset of exhaustion and increase immune responsiveness [118]. Altogether, these recent studies suggest that epigenetic deregulation of cancer cells and the TME play a key role in the regulation of the immune landscape and immune responsiveness. Moreover, since these epigenetic modifications are reversible, they highlight the potential of epigenetic therapy in improving responses to immunotherapy.

The following main research questions will be the focus of the field of immunotherapy of cancer for the next several years: Can our knowledge of how somatic alterations influence the TME help us optimize immunotherapy combinations? Are there shared themes, across cancer types, subtypes, or immune response subtypes [103] that can be exploited for improving therapeutic response? How do we harmonize biomarkers derived from different technologies to best stage a patient for IO therapy and increase the likelihood of response? Will understanding the role of epigenetic re-programming downstream of molecular alterations in tumor cells reveal new opportunities to combat cancer immune-evasion strategies?

### Take-home messages and challenges for somatic genetic contributions to CIR

- There are many tumor-intrinsic characteristics that are invariably correlated to response to ICI and composition of immune microenvironment.
- Multiple levels of molecular events at genomic, epi-genomic and regulatory stages can affect the CIR.
- The uncovering of the casual mechanistic relationships between genomic and epi-genomic events and immune composition opens the possibility to reprogramming the microenvironment and offers novel therapeutic opportunities.
- How do we deal with the molecular subtypes that define intrinsic resistance to ICI and expand CIR?

### Unanswered questions for somatic genetic contributions to CIR and strategy to meet the challenges

- Can our knowledge of how cancer-intrinsic features influence the tumor microenvironment help us optimize immunotherapy combinations?
- How do we harmonize biomarkers derived from different technologies in order to specifically tailor IO therapy for a patient and increase the likelihood of response?
- Will understanding the role of epigenetic re-programming downstream of molecular alterations in tumor cells reveal new opportunities to combat cancer immune-evasion strategies?

Recognizing that the efforts aimed at addressing somatic genetic alterations in cancer are often overlapping with germline studies for various technical and analytical reasons, the SITC task force made a decision to combine the two working groups and align their strategy that has been described in the previous section, by creating broad consortia for the accrual, analysis, interpretation and validation of identified determinants of CIR. In the upcoming second workshop on CIR to be held in Houston in September 2019, we combine de facto the two working groups and will discuss the pathways for effective functional integration.

## Transcriptional changes related to CIR

**Fig. 2:**
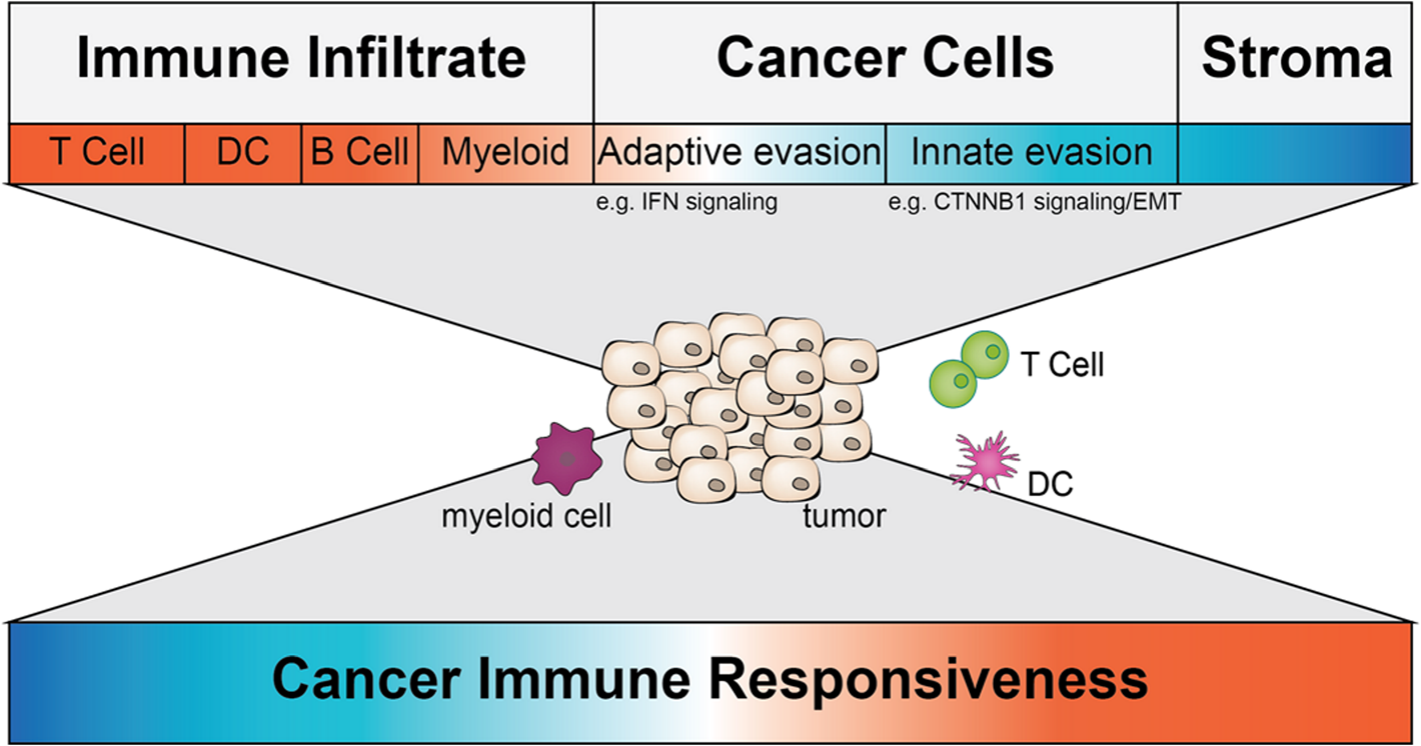
The tumor-immune microenvironment consists of a variety of cell types. All cell types comprise different transcriptional profiles. The top depicts all major categories of cell types present in a TME with a color code indicating their overall predictive value for immune responsiveness (red more responsive; blue less responsive). Some transcriptional alterations impacting immune responsiveness are highlighted beneath. The middle depicts a tumor and a subset of immune cells found within a TME and represents the challenge transcriptional profiling is facing right now. The bottom depicts the ultimate goal – using transcriptional profiling of whole tumor or single cells of the TME to predict immune responsiveness

CIR can be determined by transcriptional alterations within the TME, and transcriptional patterns can therefore be used to categorize CIR. Early studies using transcriptional profiling suggested a general association between the presence of activated, tumor-specific CD8^+^ T cells and sensitivity to immunotherapy [3, 97, 119–121]. However, with increasing understanding of the complexity of the TME [122, 123], we need to refine transcriptional analyses to include not only immune cell-derived transcripts but also transcriptional changes in cancer cells or stroma cells, which can directly influence anti-tumor immune responses (Fig. 2). The suggestion from the CIR workshop group focusing on transcriptional alterations within the TME is to separately define transcriptional alterations occurring in immune cells, cancer cells, and stromal cells (non-immune, non-cancer). While sequencing of individual cells would provide more specific information about individual contributions to the transcriptional activation of a given microenvironment, cells are generally obtained through tissue separation procedures that disrupt the spatial relationship among cells in different areas of a given tumor. Therefore, other technologies should be considered such as quantitative digital spatial profiling [124] that preserves the spatial information, contributing to the interpretation of data obtained by cell-restricted analyses.

### Immune-related transcriptional alterations

Transcriptional profiling of immune infiltrates is certainly the most advanced of the these categories with the majority of the work focusing on signatures associated with cytotoxic CD8^+^ T cell activation [3, 20, 122, 123, 125, 126]. However, even for the assessment of tumor-reactive CD8^+^ T cells, different groups have used distinct signatures. Moving forward, it will be critical to identify the specific set of genes faithfully predicting intra-tumor CD8^+^ T cell infiltration and to transcriptionally define key immune cell types directly involved in the development/modulation of T cell responses, including immune potentiating dendritic cells or immune suppressive innate cells (i.e. neutrophils, tumor-associated macrophages). While deriving a consensus transcriptional signature for CD8^+^ T cells should be achievable using currently available data sets, more effort is needed to comprehensively characterize genes involved in immune regulation of dendritic cells, macrophages, NK cells, and neutrophils before specific signatures can be generated.

Advances in single cell genomics now allow paired analysis of T-cell receptor (TCR) repertoire and transcriptional profiles associated with specific TCR clones within the TME. Identification of TCR-α and TCR-β chain pairs in concert with elucidation of neo-antigens or tumor-associated antigens might allow us to decipher the immune-dominant T cell responses across multiple patients, or across different tumors within the same patient. Initial studies in melanoma patients and melanoma-bearing mice are revealing an association between relative expansion of certain T cell clones and responses to immunotherapy [127, 128]. Analysis of the TCR repertoire of tumor-infiltrating lymphocytes (TIL) could be paired with longitudinal analysis of blood samples to elucidate whether tumor-reactive T cells can be detected in the periphery.

### Cancer cell-related transcriptional alterations

Over the last couple of years, an increasing number of preclinical and clinical studies have provided evidence that transcriptional alterations within tumor cells can have a direct impact on the abundance and functionality of immune cells within the TME [41, 47, 125]. Specifically, activation of certain oncogenic pathways, such as those controlled by beta-catenin, *epidermal growth factor receptor* (*EGFR*), *anaplastic lymphoma kinase* (*ALK*), and RAS/RAF/MEK as well as expression of mesenchymal transition genes, have been all found to contribute to immune resistance [41, 125, 129–132]. Interestingly, co-enrichment of genetic signatures corresponding to mesenchymal transition, angiogenesis, wound healing, and hypoxia in baseline tumor samples was found to identify tumors with innate resistance to anti-PD-1 therapy across different cohorts of patients with metastatic melanoma [41]. Alterations in transcription are often mediated by distinct mutations or epigenetic alterations and would therefore represent defined biomarkers predicting resistance to currently used immunotherapies [115, 133, 134]. However, thus far it is unknown whether these transcriptional alterations are cancer type-specific or not. Understanding common tumor-intrinsic features inhibiting anti-tumor immunity across cancers might be informative for the identification of broadly applicable tumor cell-intrinsic signatures mediating resistance. Importantly, these tumor-intrinsic factors can serve as both predictive and prognostic biomarkers that may improve patient selection, therapeutic decisions, and the identification of rational co-targets for more effective immunotherapy-based combinations.

### Immune-related cancer cell-intrinsic transcriptional alterations

Alterations in expression of genes associated with tumor-immune recognition have been primarily associated with both innate and acquired resistance to immunotherapy. These alterations mainly include deficiencies in antigen-presentation machinery and the IFN-γ response pathway [102, 135–137]. Loss of these functions are generally enriched in tumors characterized by elevated mutational load and T-cell infiltration, suggesting that this may be the result of an active immune escape process promoted by ongoing anti-tumor T-cell responses [20, 138]. Even though alterations in antigen-presentation machinery and the IFN-γ response pathway have been identified for the association with resistance to checkpoint blockade [101, 135, 136], it remains unclear if these alterations develop de novo in response to immunotherapy or are present at low frequency before treatment. Should the latter be the case, the development of more sensitive technologies detecting those alterations might assist in predicting acquired resistance and allow for the targeted use of combination therapies.

### Stroma-related transcriptional alterations

Similar to cancer cell-intrinsic alterations, several reports suggested that fibroblasts within the TME can interfere with anti-tumor immune responses [32, 139]. As the non-immune stroma compartment within the TME is the least-defined non-cancer compartment, transcriptional profiling should first focus on its precise characterization before engaging in mechanistic studies.

Besides refining stroma-related signatures, there is an unmet need to pair analysis of stroma transcriptional changes with immunohistochemistry or multiplex immunofluorescent staining. This integrated analysis would empower conclusions drawn from transcriptional analyses and would further increase its ultimate predictive value. To complete this integrative approach, a major undertaking to generate databases is needed to correlate the transcriptional profiles (and other patient specific information) with clinical response to immunotherapy including immune-checkpoint blockade.

Efforts to obtain a comprehensive understanding of the transcriptional profiles defining T cell-inflamed and non-inflamed TMEs and their clinical impact are further hampered by the current imprecise criteria to assess clinical responses to immunotherapy. Commonly used clinical criteria, overall survival and progression free survival, do not always capture the true nature of response at the level of the single tumor lesion. For example, while most tumor lesions in a patient might be successfully eliminated by the immunotherapeutic treatment, one individual lesion might progress. This isolated progression event would mark this patient as “resistant” to immunotherapy per response criteria but might confuse the interpretation of the underlying biology. Likely, these mixed responses represent two different biological phenomena of immune responsiveness: 1) the cancer may be intrinsically responsive to immunotherapy and 2) individual lesions may have developed escape mechanisms (acquired immune deficiency) that allows their isolated growth. Thus, mixed responses and related survival should be categorized separately as biologically closer to acquired immune resistance than other forms of lack of response that denote a non-responsiveness to the first therapy, such as primary, compensatory, and pseudo immune-resistance. Probably, it would be better to consider long-term progression-free survival and long-term overall survival, or other clinical endpoints. These parameters are less affected by conditions, which can influence the median value [140]. If the transcriptionally profiled biopsies were taken from a lesion that regressed, the analysis would correctly predict response to immunotherapy [141].

More accurate clinical assessment could significantly improve the research efforts to reveal transcriptional profiles associated with response or resistance by tracking the evolution of the biopsied lesions following immunotherapy, rather than the overall health of the patient. Likewise, a tremendous amount of information could be gained if multiple lesions within the same patients could be analyzed in order to concurrently eliminate any local or intra-patient factors impacting on anti-tumor immunity [141]. Contrasting the signature of progressing lesions to responding lesions from the same individual patients who clinically benefit from the immunotherapy may characterize their intra-tumoral heterogeneity. At best, these analyses should be done using longitudinal profiling to gain information on alterations occurring over time in response to immunotherapy. Additionally, these longitudinal analyses of individual lesions would be extremely useful for clarifying the transcriptional profiles primarily associated with primary resistance to immunotherapy or acquired resistance following an initial response.

### Take-home messages and challenges for transcriptional changes related to CIR

- Generation of transcriptional immune signature linked to functional impact of immune cells on the overall anti-tumor immunity.
- In order to understand the effects of tumor cell-intrinsic signaling on anti-tumor immunity, generate methods that allow increased resolution of tumor-immune interactions.
- Obtain longitudinal insights into how immunotherapy, and therapy in general, is impacting immune infiltration and cancer cell signaling.

### Unanswered questions for transcriptional changes related to CIR and strategy to meet the challenges

- Can we generate transcriptional signature with high predictive value for a specific tumor-immune microenvironment?
- Can transcriptional profiling be developed as a biomarker for the CIR?
- What technological advances do we need to dissect the tumor-immune microenvironment in space and time?

As per germline and somatic genetic studies, the success of this focused effort by SITC will depend on the collection and sharing of congregate information that can integrate genetic with transcriptional, clinical, and epidemiological data. In addition, other layers of potentially useful information will depend on the integration of environmental and behavioral information that may affect individual patients, such as co-morbidities, associated therapies, dietary habits, microbiome composition etc. As transcriptional information can most effectively summarize genetic with functional information, it will be the primary role of this task force to identify venues for integration and entice support from different agencies for the accrual and/or access to quality information that will be queried systematically following hypothesis-driven path. As described in this section, as much as complicated that it may appear, cancer immune biology is starting to demonstrate recognizable patterns and predictable associations with potential causative implications. Thus, we predict that more hypothesis validation, rather than discovery approaches, will help solve the enigma of CIR.

## Immunogenic cell death and CIR

**Fig. 3:**
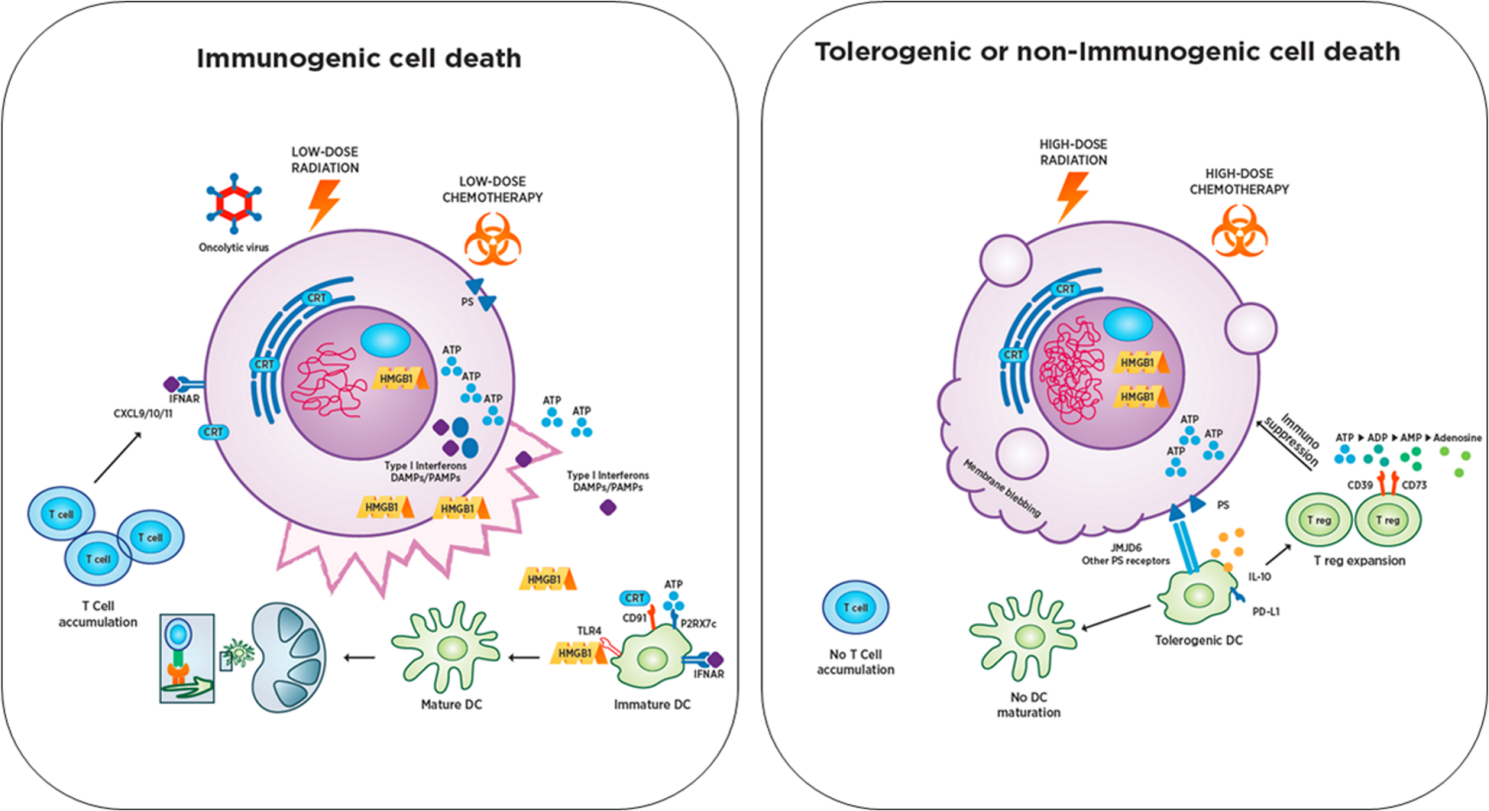
Immunogenic Cell Death (ICD) and Tolerogenic Cell Death (TCD). Immunogenic cell death can be induced by a variety of mechanisms that are still being defined, including low dose radiation, low dose chemotherapy, oncolytic viruses and others. ICD triggers translocation or release of DAMP factors from the dying cell in distinct spatiotemporal patterns that shape the subsequent immune response. DAMPs engage with receptors on *antigen presenting cells (APCs)* and, in combination with tumor-associated antigens and type I IFN, trigger APC activation, maturation, and trafficking to draining lymph nodes. This process can be augmented with TLR agonists in some instances. Once in the lymph node, APCs engage with cognate T cells and drive T cell activation and proliferation. T cells then traffic to the tumor via CXCL9/10/11 gradients induced by type I IFN signaling in tumor cells, which can result in rapid tumor elimination and generation of long term protective immune memory. In contrast, TCD including most forms of apoptosis is a non-inflammatory pathway for cell death which is characterized by membrane blebbing and loss of DAMP secretion, with sequestration of *high-mobility group protein 1 (HMGB1)* and phosphatidylserine exposure on the cell surface. Consequently, pro-inflammatory cytokines including IL-1 and TNF are not released to activate endothelium and recruit other T cells. Ectonucleotidases CD39 and CD73 degrade ICD-associated ATP to adenosine thereby inhibiting T and NK cell responses with expression of the A2A adenosine receptor (ADORA2A). This mechanism is used by *regulatory T cells (Treg)* and inhibits T cell effector function. An immunosuppressive environment characterized by enhanced myeloid derived suppressor cells and regulatory T cells is established while T cells fail to activate and form a productive immune response

Immunogenic cell death (ICD) is a regulated form of cell death accompanied by the release of DAMPs that modulate the immunogenic potential of dying cells [29, 142]. ICD is defined by its functional consequence – the generation of protective immunity specific for dying cancer cells. During early tumor progression, cancers co-exist in homeostatic balance with the immune response – immune stimulatory and immunosuppressive events permit limited immune activation but prevent complete elimination of the tumor. When tumors manifest clinically, immunosuppression mediated by regulatory T cells and myeloid-derived suppressor cells allows tumor growth to outpace immunological control. ICD shifts the immune balance from suppression to activation and enables a productive adaptive immune response coupled with long-lasting immunological memory. Thus, ICD consists of two main components: 1) tumor cells that die in a way that promotes immunity, and 2) immune cells recruited to the TME that respond and generate protective immunologic memory (summarized in Fig. 3).

### Initiation of ICD and effects on immune response

Numerous forms of regulated cell death have the potential to induce ICD, and each is characterized by unique spatiotemporal sequences of DAMP release along with release of tumor-associated antigens and recruitment of antigen presenting cells [29, 143]. In contrast to ICD, tolerogenic cell death (TCD) results in the absence of or dampening of an immune response associated with cell death (see Fig. 3). Notably, classical apoptosis does not induce ICD, favoring rather TCD, although specific variants of apoptosis as well as other forms of cell death may promote ICD [144, 145]. Tumor intrinsic factors shape the threshold for ICD based on relative levels of various signaling pathways. Similarly, extrinsic factors (including differences in the TME based on location, stage, and cellular composition) will shape both the threshold and type and quality of immune responses to ICD. Agents, which activate ICD, also affect non-malignant components of the TME, which may further shape the subsequent immune response and/or the sensitivity of the tumor to immune effectors.

### Therapeutic manipulation of ICD

The mechanism of cell death affects immunogenic potential by inducing varying patterns of DAMP release, and efficacy of ICD-inducing therapy is shaped by factors intrinsic to the cancer cells, the TME, and infiltrating immune cells. Chemotherapy and targeted therapy may induce variable quality and quantity of ICD depending on the underlying mechanism of cytotoxicity, dose of the agent used, and sensitivity of the local host immune cells to these agents. For many therapies, especially chemotherapy or radiation, lower doses may induce more potent immune responses than higher doses, with the added benefit of fewer side effects, by changing the temporal dynamics of DAMP release and cell death [146–149]. Oncolytic viruses may overcome a hostile microenvironment and recruit immune cells by selectively killing neoplastic cells and inducing high rates of soluble antigen and DAMP release while triggering type 1 IFN production to activate innate and adaptive immune responses [150]. Autophagy, a cellular response to stress that causes recycling of internal organelles for energy, may alter release of DAMPs or other immune stimulatory molecules and a cell’s sensitivity to subsequent cell death, and can impact efficacy of ICD inducing therapeutics. Heightened autophagy can induce resistance to chemo- and radiotherapy [151], whereas therapeutics modulating autophagy pathways may combine with immune checkpoint blockade to increase anti-tumor activity [152].

### Monitoring effects of ICD

There are two classical models for measuring ICD in experimental settings in vivo [153]. In the vaccination model, a cancer cell line is treated in vitro with a potentially ICD-inducing agent and inoculated into immunocompetent hosts, followed by a challenge with viable tumor cells. True ICD will induce protective immunity and tumors will not form at the challenge site. However, this model may not be suitable for all forms of ICD due to spatiotemporal differences in cell death and DAMP release. The other system for measuring ICD is an abscopal model where tumors are implanted in vivo into bilateral flanks, and a potentially ICD-inducing therapy is delivered to a single tumor site. Elimination of the untreated tumor is evidence for the generation of systemic productive immunity, an effect that has recently been defined as an ‘anamnestic’ response [154]. However, this model can only be used to investigate local therapies because treatment must be restricted to a single tumor. One of the significant challenges of monitoring ICD in vivo is the occurrence of efferocytosis, the process whereby phagocytes quickly remove dead cells and promote immunosuppression, which makes cell death difficult to confirm directly [155].

### Biomarkers of ICD

To monitor the induction of ICD and its downstream effects, a robust biomarker strategy must be incorporated routinely into clinical trials. These biomarkers should measure the type of cell death, the release of DAMPs, and the abundance, identity, and location of immune cells that contribute to a functional adaptive immune response. Specifically, biomarkers should distinguish between bona fide ICD and direct immune modulatory effects of the therapeutic drugs by focusing on cell types that are immediate sensors of DAMPs. As the end stage of ICD is a protective T cell response, T cell populations also should be measured to assess success of these strategies. Proposed clinical biomarkers of ICD include direct measurements of DAMPs such as HMGB1, ATP, *calreticulin* (*CALR*), type I IFNs, histones, *heat shock proteins* (*HSPs*), markers of immunological fitness (LDH release, soluble serum markers), and specific immune cell populations (T cell repertoire and phenotype) or cell surface receptors they express. One promising biomarker strategy is the systemic immune inflammation index, which measures ratios of specific immune cell types (e.g. neutrophils vs. lymphocytes) to monitor shifts in the balance of immune populations accumulating within the TME and peripheral circulation [156, 157]. Once biomarkers are identified, they need to be incorporated into early and late stage clinical trials to build a database of ICD in different clinical and therapeutic settings.

### Take-home messages and challenges for the ICD field

- Tumor cell death can be classified as either immunogenic (eliciting protective immunity) or tolerogenic (failure to elicit protective immunity).
- Cell death elicited therapeutically can induce the release of DAMPs which shape the subsequent immune response.
- ICD is currently measured in model systems via its functional consequences – protection from tumor challenge, but robust biomarkers for clinical utility remain undefined.

To accelerate our understanding of ICD and ability to manipulate it for clinical benefit, the field needs to:

- Characterize upstream and downstream events that drive ICD on a genetic, molecular, and cellular level.
- Establish better animal models for ICD assessment that more closely replicate human tumor immune biology.
- Develop techniques to measure tumor cell death and released DAMPs in vivo accurately and efficiently.
- Improve biomarkers to measure consequences of ICD including the induction and maintenance of anti-tumor immunity.

### Unanswered questions for the ICD field and strategy to meet the challenges

- What are the key molecular events that occur during immunogenic cell death that prime a robust immune response and promote immunological memory?
- Which therapeutic strategies will more effectively promote ICD while minimizing off target inhibition of immune responses?
- How can detection of immunogenic cell death be routinely incorporated into clinical trials?

A clear value of the study of ICD is its relevance to the determinism of CIR. The value of in vitro ICD studies is limited because several aspects of the immune physiology determining CIR can only be study in in vivo models. Yet, as discussed in the following section, animal (mouse) models have their own limitations. Thus, a decision was made by the SITC task force to integrate the two study groups into one to better define ways to exploit the induction of DAMPs in the context of ICD in xenograft models or other tissue modeling substitutes. This approach will be able to help us understand the role of different components of innate and adaptive immunity and how the components are sequentially involved in the determinism of CIR. Thus, the two working groups will be combined in the upcoming CIR workshop with the intent of building hypothesis-driven models that could address the respective questions and other related questions, for instance, the requirement of chemo-attraction and the mechanisms of chemo-repulsion that may affect migration of adoptively transferred immune cells. These issues will be the basis of discussion going forward.

## Experimental models of the immune landscape of Cancer

Despite advances in cancer genomics and cell biology to aid the rational design of new oncology drug targets, the rate of translating promising preclinical findings into successful human clinical trials remains extremely low. One obvious reason is that animal models of tumors do not reflect all of the features of genetic heterogeneity, complex tissue architecture, and immune microenvironment of human cancer. There is an urgent need to develop well-characterized preclinical models to improve the correlation between preclinical efficacy and clinical outcomes [158, 159]. To further understand the current state of the art of this topic, SITC Workshop Session V provided an overview of mouse models used in cancer immunology research and drug discovery. Special attention was focused on humanized mouse models, carcinogen-induced mouse models, and modeling of the TME.

### The advantages and limitations of currently available humanized mouse models

In past decades, there was significant advancement in the development of immune-compromised mice, from athymic nude to *Severe Combined Immune-Deficiency* (*SCID*) to *Non-obese-diabetic SCID Gamma* (*NSG*) murine models [160]. NSG mice demonstrate high engraftment efficiency of human *hematopoietic stem cells* (*HSC*), but success has been limited because of the gradual development of xenogeneic *graft-*versus*-host-disease* (*GVHD*) [161]. HSC growth and differentiation is also impaired by lack of the appropriate human cytokine expression by the host animal [160]. The lack of an intact lymphoid architecture and adaptive immunity results in failure to replicate the TME [2]. To highlight examples of humanized mouse work in immunotherapy research, a recent study of pembrolizumab efficacy in triple negative breast cancer *patient-derived xenografts* (*PDX*) in HSC-engrafted NSG mice has shown the similar response patterns as in patients [162, 163]. While these studies are promising, there is room for improvement in these models, and the development of next generation humanized mice to provide preclinical models for drug development is in progress.

### Design of the next generation of humanized mouse models

The next generation of humanized mouse models has to focus on ameliorating the deficiencies of current models. Transgenic expression of human cytokines, HLA molecules, and certain hormones would help this goal. In addition, knockdown of mouse *major histocompatibility* (*MHC*) Class I and Class II could reduce the severity of GVHD [164]. One of the major approaches for next generation humanized mouse development is to express human cytokines and other genes in the mice, such as *human macrophage colony-stimulating factor (hM-CSF*), *hIL-3*/*hGM-CSF*, *human signal regulatory protein* (*hSIRPA*), *human thrombopoietin* (*hTPO*), Rag2-deficient, γ-chain negative (*MISTRG*), and NSG mice carrying the transgenes for hstem cell factor, hGM-CSF, and *hIL-3* (*NSG-SGM3*). These new generation mice are engineered with a CSF-1-dependent and -independent compartment, enabling the study of the interaction between myeloid cells, antigen presenting cells, and T cells in the reconstituted TME [165, 166]. Melanomas implanted in MISTRG mice have enhanced primary tumor development associated with increased human macrophage infiltrate, which has also been demonstrated in human patients. NSG-SGM3 mice expressing hCSF and hGM-CSF/IL-3 transgenes have shown enhanced frequency of intra-tumoral regulatory CD4+ T cells [166].

### Leveraging carcinogen-induced mouse models to study sensitivity and resistance to cancer therapies

Despite some encouraging initial responses, ICIs are not effective in many patients with lung cancer, and acquired resistance is often implicated in clinical failure [167]. To study mechanisms of resistance, a carcinogen-induced squamous lung cancer model originally sensitive to immune checkpoint blockade has been used to mechanistically validate resistance-specific genetic alterations identified by DNA and RNA sequencing of recurring tumors from patients after treatment. For instance, *beta-2-microglobulin (B2m)* gene loss has been identified in therapy-resistant tumors [90, 101, 135, 136]. Knockout of *B2m* in the carcinogen-induced lung squamous cell line (UN-SCC680AJ) susceptible to PD-1 blockade confers resistance to PD-1 blockade [136]. Such a platform represents a useful system to validate and test tumor-cell intrinsic factors that contribute to resistance to immune checkpoint blockade. Since TMB is positively associated with response to immune checkpoint blockade, it is likely that models in which the tumors have an elevated mutational load (e.g. carcinogen-induced models) will be valuable for studying sensitivity and resistance to cancer immunotherapies. Indeed, in current mouse models, especially the genetically engineered models, the representation of the mutation loads found in human tumors is limited. Thus, improved strategies in the aspect of human/mouse discrepancy should be identified.

### Modeling of the TME

Studying the complex niche of the TME is critical for understanding key questions related to IO. For example, the lack of efficacy of adoptively transferred T cells such as *chimeric antigen receptor* (*CAR*) T cells for solid tumor in either immune active or immune silent tumors. The IO approaches and the corresponding TME models are different. Altering the balance in the immunosuppressive TME to enhance immune activation could focus predominantly on experimental models of adaptive immune responses such as the balance between Th2 and Th1. This may include blockade of CSF1-mediated sustenance of macrophages and IL-4/IL-13-mediated Th2 cell growth [168]. Based on mounting evidence that CSF1 receptor antagonists improve the efficacy of immunotherapy, the combination of anti-CSF1 receptor antibody with anti-PD-1 antibody and chemotherapy has been studied in the MMTV-PyMT breast cancer mouse model. The triple combination led to primary tumor regression and diminished pulmonary metastasis compared to monotherapy of the agents, or any two of the agents in combination (Coussens et al. unpublished data).

Another issue in preclinical models is the tissue-specific regulatory activity displayed by different types of tumors. In mesothelioma, Th2 cells promote macrophage pro-tumorigenic programing of tumor tissue and further inhibit antigen presentation by dendritic cells to CD8^+^ T cells. In squamous cell carcinoma, in contrast, B cells and humoral immunity enhance macrophage pro-tumorigenic programing and subsequent tumor growth, as well as inhibit antigen presentation to CD8^+^ T cells [169, 170]. Therefore, in squamous cell carcinoma, anti-CD20 or other relevant inhibitors, such as those targeting Bruton’s Tyrosine Kinase, could switch the TME from pro-tumorigenic to anti-tumorigenic.

### Surrogate ex vivo models to study CIR

Testing mechanisms of CIR in vivo by adopting animal models has several limitations for the reasons discussed above. Surrogate approaches have been described attempting to utilize tissue reconstructs such as three-dimensional organoids [171], in vitro three-dimensional printing [172] and other three-dimensional models [173] that have been used to assess the role of various components of the TME and potential targets for therapy including stromal matrix and proteins involved in cell-to-cell interactions by coculturing with lymphocytes [171, 173–175]. The advantage of these models is their amenability to stringent control of the tissue components assessed within a given experimental context and may in fact represent ideal grounds to test basic concepts related to migration and activation of immune cells in different conditions of immune exclusion and immune suppression. To date, however, limited experience has been gained in using these models systems to study the complex and multi-parametric biology of CIR despite emerging evidence of their utility [176–178]. For instance, Sherman et al. [178] have analyzed the chemotactic response of NK cells to human stromal-cell derived factor-1α that permits the simultaneous investigation of immune cell homing, invasion, and cytotoxic activity.

### Take-home messages and challenges in the use of experimental models

- Mouse models are important to answer mechanistic questions that are unlikely to be answered by clinical trials and to validate hypotheses generated from clinical observations. The choice of mouse model needs to be selected for the experimental purpose:
  - Relevant immune cells and cytokines need to be considered for the therapy being tested
  - If necessary, implantation site should be matched to cancer tissue of origin (orthotopic vs. subcutaneous)
  - If possible, the genetics of the tumor model should match the corresponding human genetic background status that it is supposed to represent
    - Best done with immune PDXs or genetically engineered mouse models
- While humanized mouse models are becoming more representative, challenges remain:
  - Limitations in supporting robust human immune responses need to be overcome
  - Cost remains high (tends to lower throughput)
  - There is an unmet need for unlimited HSCs for reconstitution
  - Transplantable cells may not be necessarily representative of the original tumors
- Individual models have their own limitations:
  - Transgenic models have very low mutation load and poor intra- and inter-tumor heterogeneity that may not be representative of any human tumor
  - Carcinogen models have high mutational load and high heterogeneity that may make them unrealistically immunogenic
  - PDX models may become skewed in their biology by the diverse immune environment encountered by growing in a different species
  - Fragments of PDXs maintain, at least initially, intra-tumoral immune cells but these are lost in subsequent passages making them dynamically unstable
- All immune cells should be analyzed when possible:
  - Most of the field is currently CD8^+^ T cell-focused
  - Other cell types are important in effector functions
  - APCs, myeloid, B cells, and other rare cells subtypes remain understudied

### Unanswered questions in the use of experimental models and strategy to address the challenges

- What are the current limitations of humanized PDX mouse models?
- What approaches can be undertaken towards more faithful models of human cancer-human myeloid cells interface?
- How to develop models that better model to reproduce tumor mutational load?

As described in the previous section, animal and surrogate tissue modeling system are critical to understand the physiology of innate and adaptive immune responses. No individual model suffices as it cannot represent the heterogeneity of cancer tissues from the same patient and, even more, among patients and distinct cancer typologies. In particular, the determinism of CIR can be dissected starting from basic concepts such as the dissection of the role of ICD as an initiator of the danger signal. Only context dependent models will be able to dissect this question satisfactorily and, therefore, the two conceptually overlapping working groups will be combined to address the role of ICD and innate immune activation in the most relevant model system based on a hypothesis-driven path.

## Summary and key questions from entire workshop

Over the course of the two-day workshop, five working groups of the SITC Cancer Immune Responsiveness Task Force (CIR), incorporating workshop attendees, discussed the different determinant of CIR, i.e. the genetic background of the host, somatic alterations related to the oncogenic process, and environmental modifiers, and the development of improved in vivo models for screening therapeutic strategies. Following the workshop, each working group identified the most relevant questions that will help advance the understanding of CIR (see Table 1). These key questions and scientific needs will help define priorities for research in tumor immunology and immunotherapy in order to understand the cancer biology that orchestrates distinct immune landscapes. The workshop defined the need to develop specific working groups to tackle the questions identified in this occurrence. A follow-up workshop is being organized by SITC to be held in Houston on September 4–5, 2019 that will bring the various working groups together for the delineation of the collaborative projects, and such activities will be the subject of the meeting report.

**Table 1:** Main unanswered questions identified by each working group

WORKING GROUP	Main Questions
I. Germline GENETIC Contributions TO Cancer Immune Responsiveness	1. Which are the key molecular mechanisms involved in anti-tumor immunity that might be modulated by germline genetic variants? 2. Are common genetic polymorphisms associated with a differential spontaneous or treatment-induced anti-tumor immune response? 3. How can we implement the study of host genetic diversity to identify novel biomarkers of responsiveness or toxicity to cancer immunotherapy?
II. Somatic GENETIC Contributions TO Cancer Immune Responsiveness	1. Can our knowledge of how cancer-intrinsic features influence the tumor microenvironment help us optimize immunotherapy combinations? 2. How do we harmonize biomarkers derived from different technologies in order to specifically tailor IO therapy for a patient and increase the likelihood of response? 3. Will understanding the role of epigenetic re-programming downstream of molecular alterations in tumor cells reveal new opportunities to combat cancer immune-evasion strategies?
III. Transcriptional Changes Related to CIR	1. Can we generate transcriptional signature with high predictive value for a specific tumor-immune microenvironment? 2. Can transcriptional profiling be developed as a biomarker for the CIR? 3. What technological advances do we need to dissect the tumor-immune microenvironment in space and time?
IV. Immunogenic Cell Death and Cancer Immune Responsiveness	1. What are the key molecular events that occur during immunogenic cell death that prime a robust immune response and promote immunological memory? 2. Which therapeutic strategies will more effectively promote ICD while minimizing off target inhibition of immune responses? 3. How can detection of immunogenic cell death be routinely incorporated into clinical trials?
V. Experimental Models of the Immune Landscape of Cancer	1. What are the current limitations of humanized PDX mouse models? 2. What approaches can be undertaken towards more faithful models of human cancer-human myeloid cells interface? 3. How to develop models that better model to reproduce tumor mutational load?

## Abbreviations

ACT: Adoptive Cellular Therapy
ADCC: Antibody-Dependent Cytotoxicity
ALK: Anaplastic Lymphoma Kinase
APC: antigen presenting cells
B2m: beta-2-microglobulin
CALR: Calreticulin
CAR: Chimeric Antigen Receptor
CCR: C-C motif chemokine Receptor
ccRCC: clear cell renal cell cancer
cfDNA: Cell Free DNA
CIR: Cancer Immune Responsiveness
CSF: Colony-Stimulating Factor
CTC: Circulating Tumor Cells
CTLA: Cytotoxic T-Lymphocyte-Associated protein
DAMP: Damage-Associated Molecular Pattern
DDR: DNA Damage Response
EGFR: Epidermal Growth Factor Receptor
Fc: Crystallizable Fragment
GM-CSF: Granulocyte-Macrophage Colony-stimulating Factor
GVHD: Graft-Versus-Host Disease
GWAS: Genome Wide Association Studies
HLA: Human Leukocyte Antigen
HMB1: High-Mobility group Box protein 1
HSC: Hematopoietic Stem Cell
HSP: Heat Shock Protein
ICD: Immunogenic Cell Death
ICGC: International Cancer Genome Consortium
ICI: Immune Checkpoint Inhibitor
ICR: Immunologic Constant of Rejection
IDH1: Isocitrate Dehydrogenase 1
IFN: Interferon
IL: Interleukin
IO: Immuno-Oncology
M-CSF: Macrophage Colony-Stimulating Factor
MHC: Major Histocompatibility molecules
NGS: Next Generation Sequencing
NK: Natural Killer
NSG: Non-obese-diabetic SCID Gamma mice
NSG/SGM3: NSG mice/*h*-Stem Cell Factor, *h*-Granulocyte Macrophage-Colony Stimulating Factor and *h*-IL-3 mice
PD-1: Programmed cell Death protein 1
PDX: Patient-Derived Xenograft
SCID: Severe Combined Immunodeficiency
SIRPA: Signal Regulatory Protein Alpha
SITC: Society for Immunotherapy of Cancer
TCD: Tolerogenic Cell Death
TCGA: The Cancer Genome Atlas
TCR: T-Cell Receptor
TIL: Tumor-Infiltrating Lymphocytes
TIS: Tumor Inflammation Signature
TMB: Tumor Mutational Burden
TME: Tumor Microenvironment
TPO: Thrombopoietin
Treg: Regulatory T cells
