# Correction to: Toward a comprehensive view of cancer immune responsiveness: a synopsis from the SITC workshop

**DOI:** 10.1186/s40425-019-0640-y

**Published:** 2019-07-04

**Authors:** Davide Bedognetti, Michele Ceccarelli, Lorenzo Galluzzi, Rongze Lu, Karolina Palucka, Josue Samayoa, Stefani Spranger, Sarah Warren, Kwok-Kin Wong, Elad Ziv, Diego Chowell, Lisa M. Coussens, Daniel D. De Carvalho, David G. DeNardo, Jérôme Galon, Howard L. Kaufman, Tomas Kirchhoff, Michael T. Lotze, Jason J. Luke, Andy J. Minn, Katerina Politi, Leonard D. Shultz, Richard Simon, Vésteinn Thórsson, Joanne B. Weidhaas, Maria Libera Ascierto, Paolo Antonio Ascierto, James M. Barnes, Valentin Barsan, Praveen K. Bommareddy, Adrian Bot, Sarah E. Church, Gennaro Ciliberto, Andrea De Maria, Dobrin Draganov, Winson S. Ho, Heather M. McGee, Anne Monette, Joseph F. Murphy, Paola Nisticò, Wungki Park, Maulik Patel, Michael Quigley, Laszlo Radvanyi, Harry Raftopoulos, Nils-Petter Rudqvist, Alexandra Snyder, Randy F. Sweis, Sara Valpione, Roberta Zappasodi, Lisa H. Butterfield, Mary L. Disis, Bernard A. Fox, Alessandra Cesano, Francesco M. Marincola, Society for Immunotherapy of Cancer (SITC) Cancer Immune Responsiveness Task Force and Working Groups

**Affiliations:** 1 Sidra Medicine, Doha, Qatar; 2 AbbVie, Redwood City, CA USA; 3 Department of Radiation Oncology, Weill Cornell Medical College, New York, NY USA; 4 Sandra and Edward Meyer Cancer Center, New York, NY USA; 5 Université Paris Descartes/Paris V, Paris, France; 6 The Jackson Laboratory for Genomic Medicine, Farmington, CT USA; 7 Koch Institute for Integrative Cancer Research at MIT, Cambridge, MT USA; 8 NanoString Technologies, Inc, Seattle, WA USA; 9 Perlmutter Cancer Center, New York Langone Health, New York, NY USA; 10 University of California, San Francisco, CA USA; 11 Memorial Sloan Kettering Cancer Center, New York, NY USA; 12 Oregon Health & Science University, Portland, OR USA; 13 Department of Medical Biophysics, Princess Margaret Cancer Centre University Health Network, University of Toronto, Toronto, Canada; 14 Washington University School of Medicine in St. Louis, St. Louis, MO USA; 15 INSERM, Laboratory of Integrative Cancer Immunology, Equipe Labellisée Ligue Contre le Cancer, Sorbonne Université, Sorbonne Paris Cité, Université Paris Descartes, Université Paris Diderot; Centre de Recherche des Cordeliers, F-75006 Paris, France; 16 Massachusetts General Hospital, Boston, MA, USA and Replimune, Inc, Woburn, MA USA; 17 Perlmutter Comprehensive Cancer Center, New York University School of Medicine, New York University Langone Health New York, New York, NY USA; 18 UPMC Hillman Cancer Center, University of Pittsburgh, Pittsburgh, PA USA; 19 University of Chicago, Chicago, IL USA; 20 Abramson Family Cancer Research Institute, University of Pennsylvania, Philadelphia, PA USA; 21 Yale School of Medicine, New Haven, CT USA; 22 The Jackson Laboratory Cancer Center, Bar Harbor, ME USA; 23 R. Simon Consulting, Potomac, MD USA; 24 Institute for Systems Biology, Seattle, WA USA; 25 University of California, Los Angeles, Los Angeles, CA USA; 26 MedImmune, Gaithersberg, MD USA; 27 Istituto Nazionale Tumori-IRCCS Fondazione ‘G. Pascale’, Naples, Italy; 28 Stanford University, Stanford, CA USA; 29 Rutgers University, New Brunswick, NJ USA; 30 Kite, a Gilead Company, Santa Monica, CA USA; 31 IRCCS Istituto Nazionale Tumori Regina Elena, Rome, Italy; 32 Università degli Studi di Genova and Ospedale Policlinico San Martino IRCCS, Genoa, Italy; 33 Calidi Biotherapeutics, San Diego, CA USA; 34 Department of Neurosurgery, Division of Pediatric Neurosurgery, Primary Children’s Hospital, University of Utah, Salt Lake City, UT USA; 35 Department of Radiation Oncology, Icahn School of Medicine at Mount Sinai, New York, NY USA; 36 Lady Davis Institute for Medical Research, Jewish General Hospital, Montreal, QC Canada; 37 Caprion Biosciences Inc, Montreal, QC Canada; 38 Bristol-Myers Squibb Company, Redwood City, CA USA; 39 Ontario Institute for Cancer Research, Toronto, Ontario Canada; 40 Bayer HealthCare Pharmaceuticals Inc, Whippany, NJ USA; 41 Merck & Co, Kenilworth, NJ USA; 42 CRUK Manchester Institute and The Christie NHS Foundation Trust, The University of Manchester, Manchester, UK; 43 Parker Institute for Cancer Immunotherapy, San Francisco, CA USA; 44 University of Washington, Seattle, WA USA; 45 Earle A. Chiles Research Institute, Robert W. Franz Cancer Center, Providence Cancer Institute, Portland, OR USA; 46 Refuge Biotechnologies Inc, 1505 Adams Drive, Suite D, Menlo Park, CA 94025 USA; 47 Ludwig Collaborative and Swim Across America Laboratory, Memorial Sloan Kettering Cancer Center, New York, NY USA; 48 Parker Institute for Cancer Immunotherapy, Memorial Sloan Kettering Cancer Center, New York, NY USA

**Correction to: J Immuno Ther Cancer**

**https://doi.org/10.1186/s40425-019-0602-4**

Following publication of the original article [1], the author reported that an author name, Roberta Zappasodi, was missed in the authorship list.

The correction has been implemented in the original article as well.

The publisher apologized for any inconvenience this might has caused.
